# Predictors of exacerbation frequency in chronic obstructive pulmonary disease

**DOI:** 10.1186/2047-783X-19-18

**Published:** 2014-04-08

**Authors:** Hui Yang, Pingchao Xiang, Erming Zhang, Wei’An Guo, Yanwei Shi, Shuo Zhang, Zhaohui Tong

**Affiliations:** 1 Department of Respiratory and Critical Care Medicine, Beijing Institute of Respiratory Medicine, Beijing Chao-Yang Hospital Beijing, Capital Medical University, No. 8 Gong Ti Southern Road, Chao Yang District, Beijing 100020, China; 2 Department of Respiratory and Critical Care Medicine, Shou-Gang Hospital Affiliated to Peking University, No.9 Jin Yuan Village Road, Shi Jing Shan District, Beijing 100041, China

**Keywords:** Chronic obstructive pulmonary disease, Exacerbation, Forced expiratory volume, Comorbidity, Positive pressure ventilation

## Abstract

**Background:**

Exacerbations of chronic obstructive pulmonary disease (COPD) are sporadic, acute worsening of symptoms. Identifying predictors of exacerbation frequency may facilitate medical interventions that reduce exacerbation frequency and severity. The objective of this study was to determine predictors of exacerbation frequency and mortality.

**Methods:**

A total of 227 COPD patients were enrolled in a prospective clinical study between January 2000 and December 2011. Reported exacerbations were recorded for the year preceding enrollment and annually thereafter, and patients were grouped by median annual exacerbation frequency into those experiencing infrequent exacerbations (less than one exacerbation annually) and frequent exacerbations (one or more exacerbation annually). Patients experiencing frequent exacerbations were further divided into those experiencing moderately frequent exacerbations (fewer than two exacerbations per year) and severely frequent exacerbations (two or more exacerbations per year). The rate of clinical relapse and survival was recorded over a 10-year period. The mean of follow-up time was 5.15 years per patient.

**Results:**

For patients experiencing infrequent, moderately frequent, and severely frequent exacerbations, median exacerbations in the year preceding enrollment were 0.0, 0.5, 1.0, respectively, and more frequent exacerbations correlated with lower baseline forced expiratory volume in one second (FEV_1_) (0.81 L, 0.75 L, and 0.66 L, respectively), higher comorbidity (70.7%, 75.0%, and 89.4%, respectively), and greater NPPV use during hospitalization (16.4%, 35.9% and 51.1%, respectively). FEV_1_ declined and mortality increased with increasing exacerbation frequency.

**Conclusions:**

Exacerbation frequency can be used to generate discreet patient subpopulations, supporting the hypothesis that multiple COPD phenotypes exist and can be used in patient risk stratification.

## Background

Chronic obstructive pulmonary disease (COPD), characterized by difficulty breathing due to chronic bronchitis or emphysema, is one of the most common lung conditions observed in clinical practice, the fourth leading cause of death in the world [1]. An exacerbation of COPD is defined as acute worsening in the severity of a patient’s symptoms, including baseline dyspnea and cough, that warrants a change in medication or medical intervention [2]. Exacerbations have been linked to disease-associated morbidity and mortality, placing significant strain on medical facilities, increasing resource burden, and driving up ongoing healthcare costs [3]. To better allocate healthcare resources, exacerbations can be classified by symptom type and severity, and by the frequency of incidents requiring intervention [4].

Exacerbation frequency varies significantly among patients [5,6], and more frequent exacerbations are linked to more rapidly declining lung function and higher mortality [5]. The importance of exacerbation prediction has recently been recognized in the application of effective and aggressive prevention strategies [3], including antibiotic therapies that are designed to reduce exacerbation frequency and severity [7]. While major advances have been made in the past decade, measureable prognostic factors predicting exacerbation risk in COPD patients remain undefined.

The most reliable predictor of increasing exacerbation frequency is conventionally considered to be a patient’s previous history of exacerbations [8], potentially indicating a definable phenotype of exacerbation susceptibility. Most published research on COPD exacerbations are large interventional studies [9,10] with highly variable exacerbation definitions [8-10]. Thus, few data have been collected that can reliably indicate clinically measurable prognostic risk factors for COPD exacerbation frequency and associated clinical outcomes.

To better assess the prognostic factors associated with COPD frequency, we followed a 227-member cohort of COPD patients over a 10-year period. We identified predictors of exacerbation frequency and generated a mechanism for improved clinical classification of COPD patients by exacerbation frequency. This classification may improve the efficacy of strategically applied prophylactic interventions.

## Methods

### Study design and patient groups

A total of 227 COPD patients were recruited consecutively between 1 January 2000 and 31 December 2006 for inclusion in a study of exacerbation frequency ranging from the inclusion start date to 31 December 2011 at the Outpatient Department of Respiration of the Shougang Hospital of Beijing University (Beijing, China). The study protocol was approved by the Medical ethics committee of Shou-Gang Hospital Affiliated to Peking University. All patients provided written informed consent for participation.

### Inclusion and exclusion criteria

Inclusion criteria included a COPD diagnosis, defined as a forced expiratory volume in 1 second (FEV_1_) of < 80% of the predicted value after bronchodilator use and a ratio of FEV_1_ to forced vital capacity (FVC) of < 70% after bronchodilator use. We recruited participants between the ages of 45 and 85 years who were in a stable condition, having experienced no exacerbations in the month preceding enrollment, and who could provide medical records indicating the number of times in the preceding year they had been hospitalizations as a result of COPD. Patients who did not consent to long-term follow-up or were diagnosed with another condition that limited life expectancy were excluded from the analysis.

### Exacerbations

**Figure 1:**
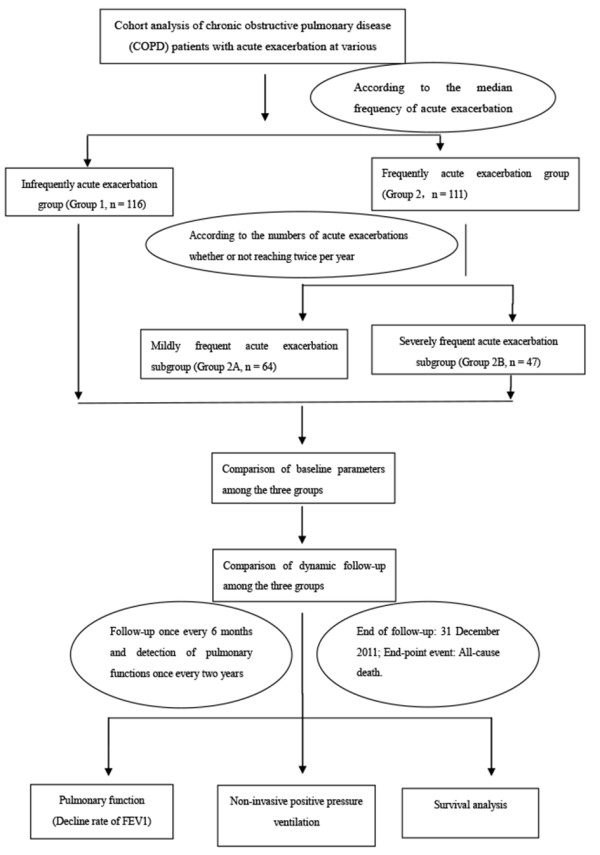
Flow diagram of study design and patient grouping.

We defined an exacerbation as sustained worsening of respiratory symptoms, such as breathlessness or increased sputum volume or purulence beyond the basal variability and that required treatment with oral corticosteroids or antibiotics [11,12]. In this study we recorded only those exacerbations resulting in hospitalization, and exacerbations separated by ≥ 14 days were considered distinct events [13-16]. Reported exacerbations were recorded for the year preceding enrollment, and annually thereafter until the end of the study or death. Patients were grouped by the median annual exacerbation frequency into those experiencing infrequent exacerbations (Group 1: less than one exacerbation annually) and frequent exacerbations (Group 2: one or more exacerbation annually). Patients experiencing frequent exacerbations were further divided into those experiencing moderately frequent exacerbations (Group 2A: fewer than two exacerbations per year) and severely frequent exacerbations (Group 2B: two or more exacerbations per year) (Figure 1).

### Data collection and follow-up

During initial clinical examinations, patient demographics were recorded, including sex, age, height, weight, body mass index (BMI), and smoking history. Clinical characteristics were recorded for each patient, including lung function, drug history (including bronchial-dilating agents, glucocorticoids), comorbidities, number of hospitalization as a result of COPD exacerbations in the year preceding enrollment, and every year throughout the follow-up period, and use of long-term oxygen therapy (LTOT), home mechanical ventilation (HMV) used for chronic hypercapnia of COPD, and non-invasive positive pressure ventilation (NPPV, used for exacerbations accompanied by acute respiratory failure) during hospitalization. Follow-up was conducted by telephone interview and review of appropriate medical records every 6 months until the end of the study. Quint *et al.* have previously determined that patient recall was a sufficiently accurate tool for stratification of patients into those experiencing frequent and infrequent exacerbations [16].

Lung function was assessed using a volumetric storage spirometer (HP 47-804; Hewlett-Packard, Waltham, MA, USA) to measure arterial carbon dioxide tension (PaCO_2_); arterial oxygen tension (PaO_2_); forced expiratory volume in one second (FEV_1_); and forced vital capacity (FVC) at baseline (year 0) and thereafter at 2-year intervals for the first 4 years. GOLD grades were calculated according to the Global Initiative for Chronic Obstructive Lung Disease.

At the end of follow-up, the Charlson comorbidity index was computed and analyzed. The Charlson Index assigns a score to each underlying condition proportional to its corresponding disease-related risk of death [17]. The arithmetical sum of scores for individual comorbidities was used as an index for each patient.

### Statistical analysis

Statistical analyses were performed using SPSS version 17.0 (SPSS Inc., Chicago, IL, USA). Continuous variables were presented as means ± standard deviation (SD) or as medians and interquartile ranges by data distribution. One-way ANOVA was used to compare exacerbation frequency. Chi-square tests with Pearson’s, continuity correction, or Fisher’s Exact test were used for binary variables. Logistic regression analysis was used to identify independent predictors of frequent exacerbations. Univariate survival analysis was performed using Kaplan-Meier analysis (log-rank test). Multivariate COX proportional hazards regression models were used to identify independent predictors of mortality. Relative risk and 95% confidence intervals (CIs) were presented. *P* values less than 0.05 were considered statistically significant.

## Results

### Clinical and demographic characteristics

A total of 227 patients were enrolled. The mean age of participants was 71.7 ± 6.8 years, and ranged from 45 to 85 years. Of these participants, 70% were male. Patients were grouped by the median annual exacerbation frequency. A total of 51.1% (116/227) of the patients experienced infrequent exacerbations (less than one exacerbation annually) and were assigned to Group 1, and 48.9% (111/227) experienced frequent exacerbations (one or more exacerbations annually) were assigned to Group 2. Patients experiencing frequent exacerbations were further subdivided by exacerbation frequency. Of these, 28.2% (64/227) experienced moderately frequent exacerbations (fewer than two exacerbations per year) and were assigned to Group 2A. Another 20.1% (47/227) experienced severely frequent exacerbations (two or more exacerbations per year) and were assigned to Group 2B (Table 1).

**Table 1:** Baseline of clinical and demographic characteristics among groups according to exacerbation frequency

**Parameters**	**Group 1 (*n*** **= 116)**	**Group 2A (*n*** **= 64)**	**Group 2B (n = 47)**	**Total (n = 227)**
Age (mean ± SD, yrs)	71.78 ± 6.36	71.02 ± 7.24	72.19 ± 7.30	71.65 ± 6.80
Male, %	73.3	67.2	66.0	70.0
Height (mean ± SD, cm)	169.6 ± 2.32	171.2 ± 3.19	170.7 ± 2.56	170.9 ± 3.03
Weight (mean ± SD, kg)	67.06 ± 4.10	67.60 ± 3.78	71.20 ± 3.98	68.90 ± 2.75
BMI (mean ± SD, kg/m^2^)	23.58 ± 3.31	23.05 ± 4.30	24.42 ± 3.68	23.60 ± 3.70
Current smoker, %	56.0	57.8	66.0	58.6
Numbers of preceding year exacerbation^a^	0 (0 to 1.0)	0.5 (0 to 2.0)	1.0 (0 to 2.0)	0 (0 to 1.0)
GOLD grade				-
GOLD 1, %	4.3	6.3	0	4.0-
GOLD 2, %	16.4	9.4	8.5	12.8-
GOLD 3, %	45.7	45.3	57.4	48.0-
GOLD 4, %	33.6	39.1	34.0	35.2-
FEV_1_ (mean ± SD, L)^b^	0.81 ± 0.36	0.75 ± 0.33	0.66 ± 0.29	0.76 ± 0.34-
FEV_1_ (mean ± SD, % predicted)	38.76 ± 16.90	37.61 ± 17.08	34.49 ± 11.91	37.55 ± 16.07-
FVC, (mean ± SD, L)^c^	1.50 ± 0.54	1.42 ± 0.49	1.27 ± 0.53	1.43 ± 0.53-
pH (mean ± SD)	7.40 ± 0.04	7.40 ± 0.04	7.40 ± 0.04	7.40 ± 0.04-
PaCO_2_ (mean ± SD, mmHg)^d^	48.03 ± 9.97	51.35 ± 10.45	49.78 ± 12.04	49.33- ± 10.61
PaO_2_ (mean ± SD, mmHg)	67.42 ± 11.67	67.10 ± 14.30	65.56 ± 13.01	66.94 ± 12.70-
Cor pulmonale, %^e^	34.5	54.7	63.8	46.3-
Comorbidity, %^f^	70.7	75.0	89.4	75.8-
Cardiovascular disease, % (n)	62.9 (73)	79.7 (51)	74.5 (35)	70.0 (159) -
Cerebrovascular disease, % (n)	28.4 (33)	23.4 (15)	36.2 (17)	28.6 (65) -
Diabetes mellitus, % (n)	14.7 (17)	14.1 (9)	12.8 (6)	14.1 (32) -
Neoplasms, % (n)	7.8 (9)	7.8 (5)	10.6 (5)	8.4 (19) -
Osteoporosis, % (n)	2.6 (3)	3.1 (2)	4.3 (2)	3.1 (7) -
Arrhythmia, % (n)	2.6 (3)	1.6 (1)	4.3 (2)	2.6 (7) -
Total comorbidity	1 (0 to 3.0)	1 (0 to 4.0)	1 (0 to 3.6)	1 (1.0-2.0) -
Charlson index (mean ± SD)^g^	5.30 ± 1.77	5.33 ± 1.94	5.62 ± 1.53	5.37 ± 1.77-
Medicine at home, %	29.3	40.6	31.9	33.0-
LTOT at home, %	6.9	15.6	10.6	10.1-
HMV, %	1.7	4.7	2.1	2.6-
NPPV in hospitalization, %*^h^	16.4	35.9	51.1	29.1-

BMI, body mass index; FEV_1_, forced expiratory volume in one second; FVC, forced vital capacity; HMV, home mechanical ventilation; LTOT, long-term oxygen treatment; NPPV, non-invasive positive pressure ventilation; PaCO_2_, arterial carbon dioxide tension; PaO_2_, arterial oxygen tension.

^a^:*P*_group 1-group 2B_ and *P*_group 1-group 2A_ < 0.001;

^b^:P_group 1-group 2B_ = 0.012;

^c^:*P*_group 1-group 2B_ = 0.013;

^d^:*P*_group 1-group 2B_ = 0.044;

^e^:*P*_group 1-group 2A_ = 0.008; and *P*_group 1-group2B_ = 0.001;

^f^:*P*_group 1-group 2B_ = 0.011;

^g^:*P*_group 1-group2B_ = 0.014;

^h^:*P*_group 1-group 2A_ = 0.003; and *P*_group 1-group 2B_ < 0.001.

The mean follow-up time was 5.15 years per patient. The number of exacerbations in the year before recruitment into this study, FEV_1_, FVC, PaCO_2_, cor pulmonale, comorbidity, and the Charlson Index score of patients varied significantly between groups (*P* < 0.05, Table 1).

### Factors associated with increased exacerbation frequency

More frequent exacerbations were significantly associated with lower FEV_1_ and FVC, a higher frequency of exacerbation in the preceding year, higher PaCO_2_, higher incidence of cor pulmonale, and a higher rate of comorbidities (*P* < 0.05, Table 1). The most common comorbidity was cardiovascular disease, though cerebrovascular disease, diabetes mellitus, and neoplasm were also observed.

### Scoring assessments indicate exacerbation frequency

**Figure 2:**
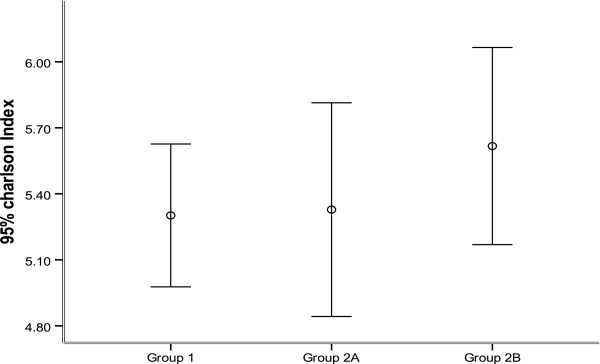
**Comparison of Charlson Index among COPD patients in Groups 1, 2A and 2B, experiencing infrequent, mildly frequent and severely frequent exacerbations respectively.** Note: p_group 1-group2B_ = 0.014.

**Figure 3:**
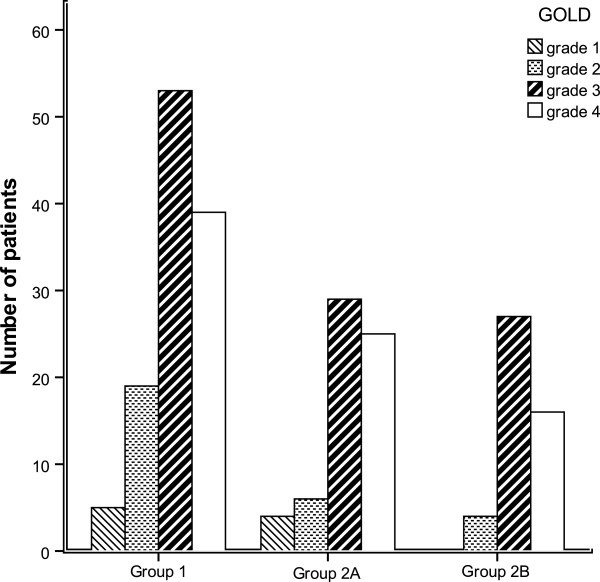
GOLD (Global Initiative for Chronic Obstructive Lung Disease) grades of COPD patients in Group 1, 2A and 2B, experiencing infrequent, mildly frequent and severely frequent exacerbations respectively.

Significantly higher Charlson Index scores were observed in Group 2B than in both Group 1 and Group 2A (*P* = 0.014, Figure 2). There was no significant difference in GOLD grades between the three groups (*P* > 0.05, Figure 3). All GOLD grades were represented in each group, with the exception that Group 2b contained no patients with a Gold grade of 1. For GOLD grades 1 to 3, the number of exacerbations increased significantly in all three groups (*P* < 0.05, Figure 3). Notably, patients with a GOLD grade of 4 experienced a significantly lower frequency of exacerbations than patients with a GOLD grade of 3 (*P* < 0.05, Figure 3).

### Comorbidity and exacerbation frequency

The proportion of comorbidities in patients assigned a GOLD grade of 4 was significantly lower that of patients with GOLD grades 1, 2 and 3 (63.8% versus 100%, 82.8% and 80.7%, respectively, all *P* < 0.05). Increased exacerbation frequency was significantly associated with higher rates of comorbidities (*P* < 0.05, Table 2).

**Table 2:** Logistic analysis of predictors for severely frequent exacerbations (Group 2B)

	**B**	**Wald**	**Sig.**	**RR**	**95% CI for RR**	
					**Lower**	**Upper**
Comorbidity	1.340	5.453	0.020	3.818	1.240	11.752
NPPV	1.145	7.691	0.006	3.143	1.399	7.062
FEV_1_	-0.954	5.096	0.024	0.385	0.168	0.882
Exacerbations in the year preceding enrollment	0.861	13.881	0.000	2.366	1.504	3.722

RR, relative risk; NPPV, non-invasive positive pressure ventilation; FEV_1_, forced expiratory volume in one second.

The rate of NPPV use by GOLD 4 patients (40.8%) was significantly higher than the rate of NPPV use by GOLD 3 (28.4%) or GOLD 2 (10.3%) patients (all *P* < 0.05). NPPV use in Group 2A (35.9%) and Group 2B (51.1%) was significantly higher than NPPV use in Group 1 (16.4%, OR 2.864, 95% CI 1.410 to 5.819, and OR 5.327, 95% CI 2.507 to 11.326, respectively, Table 2). Increased exacerbation frequency was significantly associated with higher levels of NPPV use (*P* < 0.05, Table 2).

### Forced expiratory volume in one second is related to exacerbation frequency

**Figure 4:**
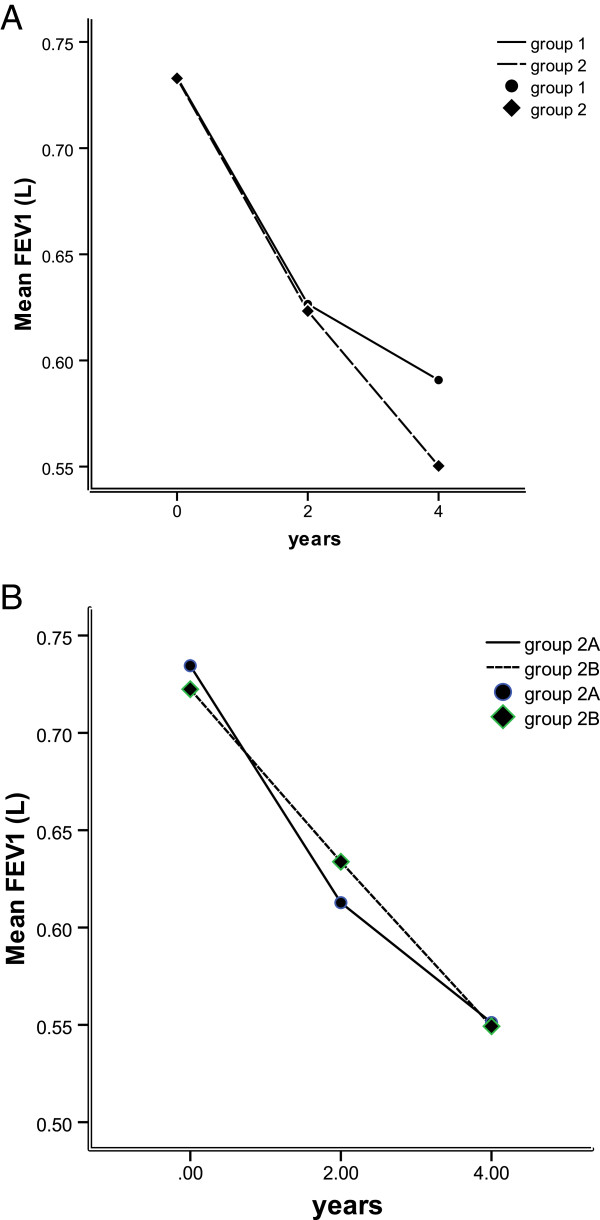
**Change in FEV**_**1**_**.** FEV_1_**(A)** in patients experiencing infrequent (Group 1) and frequent exacerbations (Group 2, including Group 2A and Group 2B) and **(B)** and those experiencing mildly frequent exacerbations (Group 2A) and severely frequent exacerbations (Groups 2B) over time.

Logistic analysis revealed that increased exacerbation frequency was significantly associated with worsening lung function, measured by post-bronchodilator FEV_1_ (*P* < 0.05, Table 2). Over the 4 years of follow-up, the FEV_1_ of patients in Group 2A and 2B declined faster than in Group 1, with the most notable decline occurring in the last 2 years (Figure 4A). Patients in Group 2B experienced consistent decline over the 4 years, compared with Group 2A patients, who experienced more rapid decline in the first 2 years of the study (Figure 4B), suggesting discreet stages of disease progression. Overall, FEV_1_ was reduced in Groups 1, 2A, and 2B by an average of 38 mL, 46 mL, and 44.6 mL per year respectively, representing no significant variation between groups (*P* > 0.05).

### Exacerbation history is related to exacerbation frequency

A history of previous exacerbations was also significantly associated with increased exacerbation frequency (*P* < 0.05, Table 2).

### Mortality increases with increasing exacerbation frequency

**Figure 5:**
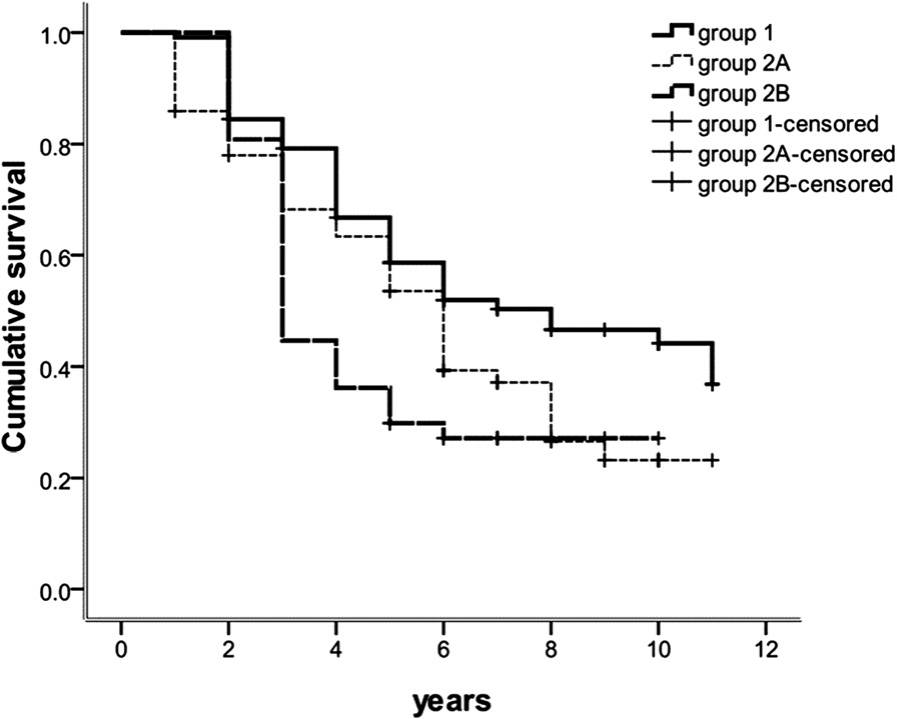
**Survival by year in COPD patient group 1, 2A and 2B, experiencing infrequent, mildly frequent and severely frequent exacerbations respectively.** Note: *P*_group 1 – group 2_ = 0.026, *P*_group 1 – group 2B_ = 0.009, *P*_group 2 A– group 2B_ = 0.561.

A total of 135 (59.5%) patients died during the follow-up window, and 2 (0.01%) patients were lost to follow-up. By the endpoint of the study, the overall mortality was 50.0%, in Group 1, 67.2%, in Group 2A, and 72.3% in Group 2B, but did not differ significantly between the three groups. The cumulative survival time of Group 1 was significantly longer than that of Group 2A and 2B, but did not differ significantly between Group 2A and 2B (Figure 5).

Deaths were due to respiratory failure (51.1%), comorbidities (46.7%), and unknown factors (2.2%). Of those deaths related to comorbidities (63 in total), 38.1% were related to infections, 25.4% were related to neoplasms, including lung cancer (62.5%), cerebrovascular disease (15.9%), cardiovascular disease (14.3%), and pulmonary embolism (6.3%).

Patients in Group 2B and 2A had mortality rates approximately 2.6 fold and 2.0 fold greater, respectively, than patients in Group 1 (95% CI 1.253 to 5.457; 95% CI 1.084 to 3.868). Significantly different 5- and 10-year cumulative survival rates were observed in Groups 1 (58% and 41%), 2A (58% and 41%) and 2B (29% and 23%) (*P* = 0.003, Table 1). More patients in Group 1 died as a result of a comorbidity than respiratory failure (51.7% versus 39.7%), whereas the majority of patients in group 2A died of respiratory failure (60.5% versus 34.9%). Patients in Group 2B died of respiratory failure and comorbidities at a similar rate (50.0% versus 44.1%).

### Verifying the prognostic factors for mortality

COX analysis by age, sex, smoking, BMI, PH, PaCO_2_, PaO_2_, FEV_1_, FEV_1_%, FVC, cor pulmonale, comorbidity, total comorbidities, Charlson Index, NPPV, exacerbations in the year preceding the study and exacerbation frequency preceding enrollment provided verification that higher rates of exacerbation frequency in the past are an independent prognostic factor of exacerbation frequency in the future (Table 3). In addition to variable exacerbation frequency, this analysis also indicated that FEV_1_, age, cor pulmonale, BMI, Charlson Index, total comorbidities, and NPPV use were also independent prognostic factors for exacerbation frequency (Table 3). However, as NPPV is administrated during the hospitalization for acute exacerbation of chronic obstructive pulmonary disease with acute respiratory failure, increased frequency of acute exacerbation resulted in increased administration of NPPV, and NPPV is not a predictor of acute exacerbation frequency.

**Table 3:** Cox analysis of independent prognostic factors for all chronic obstructive pulmonary disease (COPD) patients

**Items**	**B**	**Wald**	**Sig.**	**RR**	**95.0% CI for RR**	
					**Lower**	**Upper**
Exacerbation frequency	0.418	11.868	0.001	1.518	1.197	1.926
FEV_1_% predicted	-0.015	4.412	0.036	0.985	0.972	0.999
Charlson Index	0.201	7.311	0.007	1.222	1.057	1.413
Age	0.034	4.147	0.042	1.035	1.001	1.070
NPPV	-0.664	8.578	0.003	0.515	0.330	0.803
P_a_CO_2_	0.022	4.373	0.037	1.023	1.001	1.044
Cor pulmonale	0.390	3.988	0.046	1.477	1.007	2.165
BMI	-0.087	11.383	0.001	0.917	0.872	0.964
Total comorbidities	-0.297	7.790	0.005	0.743	0.603	0.915

RR, relative risk; FEV_1_, forced expiratory volume in one second; NPPV, non-invasive positive pressure ventilation; PaCO_2_, arterial carbon dioxide tension; BMI, body mass index.

## Discussion

A group of COPD patients that were susceptible to multiple exacerbations was identified and the associations between these occurrences and potential prognostic factors including FEV_1_, exacerbation history, NPPV use, and comorbidities were determined. Logistic analysis of exacerbation frequency during a follow-up period confirmed that the rate of exacerbations reported in the year preceding enrollment was an independent predictor of more frequent exacerbations in the future. Patients with moderately frequent and severely frequent exacerbations had mortality rates approximately 2.6 fold and 2.0 fold greater, respectively, than those experiencing infrequent exacerbations, and COX analysis confirmed that exacerbation frequency was an independent prognostic factor for elevated mortality. These findings highlight the need for improved identification and stratification of COPD patients with frequent exacerbations for more in-depth monitoring and treatment with more aggressive therapeutic strategies.

The study of COPD exacerbations is complicated by the varying definition of exacerbations employed. Studies have applied a variety of cutoffs related to the median number of exacerbations per study group [3,13,18], a relative measurement that makes it difficult to compare data between studies. In an attempt to mediate these discrepancies, some studies have applied a fixed model for assessing exacerbation frequency, wherein ‘frequent exacerbations’ are defined as those occurring two or more times a year [10,19]. This model was applied in this study, and additionally, we specified that the exacerbation events must be separated by 14 days and require hospitalization.

Several models for the prediction of COPD exacerbations have been explored over the past decade. Hurst *et al*. [6] reported that a reliable predictor of exacerbations across all GOLD stages was a previous history of exacerbations. Numerous additional studies have confirmed that previous COPD exacerbations are a risk factor for new exacerbations, independent of COPD severity and other clinical manifestations [3,5,7,8]. Similarly, within the population we studied, exacerbation frequency preceding enrollment was found to be an independent predictor of exacerbation frequency during the follow-up period.

We have identified some additional prognostic factors that can be used to stratify COPD patient risk; FEV_1_, comorbidities, and NPPV use, along with exacerbation history, were identified as independent predictors of elevated exacerbation frequency. Similarly, the TORCH (Toward a Revolution in COPD Health) trial [20] reported a negative association between exacerbation frequency and declining FEV_1_, and David *et al*. reported that higher exacerbation frequency was associated with significantly lower baseline post-bronchodilator FEV_1_. Conversely, Kirby *et al*. [21] determined that FEV_1_ was not a significant clinical predictor of frequent exacerbations. We can conclude that the number of exacerbations does not increase linearly with FEV_1_ decline, but is influenced by multiple factors.

Clinical manifestations of systemic inflammation are often observed in parallel with COPD exacerbations [22]. Extra-pulmonary factors may play a role in COPD-related declining lung function. As reported for cardiovascular exacerbations, patients with higher numbers of concomitant disorders may be more prone to exacerbations leading to frequent hospitalization [23]. These findings were confirmed in a case–control study of severe COPD patients that indicated patients with frequent exacerbations also experienced more frequent cardiovascular comorbidities [24], consistent with the elevated rate of cardiovascular and other comorbidities reported for patients experiencing frequent exacerbations in this study. However, because these observations were not found to be statistically significant, further study will be required to verify this association.

While this report identifies the association between several prognostic factors and increased exacerbation frequency in COPD patients, further study is required before broad clinical recommendations can be made.

## Conclusions

A history of frequent exacerbations, lower FEV_1_, and multiple comorbidities were independent prognostic factors for exacerbation frequency, in a 10-year study of 227 COPD patients. Patients who experienced two or more exacerbations annually were at a greater risk for early mortality than patients with less frequent exacerbations. Thus, these patients should be considered candidates for aggressive monitoring and therapy to mediate COPD disease progression and to reduce exacerbation frequency and severity. Furthermore, the stratification of patients in this study supports the hypothesis that discreet phenotypes exist that can be used to determine patient risk in clinical settings.

## Abbreviations

BMI: Body mass index; COPD: Chronic obstructive pulmonary disease; FEV1: Forced expiratory volume in one second; FVC: Forced vital capacity; GOLD: Global Initiative for Chronic Obstructive Lung Disease; HMV: Home mechanical ventilation; LTOT: Long-term oxygen therapy; NPPV: Non-invasive positive pressure ventilation; PaCO2: Arterial carbon dioxide tension; PaO2: Arterial oxygen tension.

## Competing interests

The authors declare that they have no competing interests.

## Authors’ contributions

HY, PX carried out the studies, participated in data collection, and drafted the manuscript. ZT performed the statistical analysis and participated in its design. EZ, WG, YS, SZ helped to draft the manuscript. All authors read and approved the final manuscript.
